# Different doses of ovalbumin exposure on dendritic cells determine their genetic/epigenetic regulation and T cell differentiation

**DOI:** 10.18632/aging.104145

**Published:** 2020-11-24

**Authors:** Ying Wang, Zizhong Yu, Yue Zhou, Yun Zhu, Jinhui Wang, Junmei Fu, Yang Yuan, Shan Chen, Yanjun Wang, Wenting Yu, Pei Gao, Wanting Zhu, Qing Cheng, Seong H Cho, Weijia Kong, Jianjun Chen

**Affiliations:** 1 Department of Otorhinolaryngology, Union Hospital, Tongji Medical College, Huazhong University of Science and Technology, Wuhan, China; 2 Department of Otorhinolaryngology, Taihe Hospital, Hubei University of Medicine, Wuhan, China; 3 Department of Otorhinolaryngology, First Hospital of Handan, Wuhan, China; 4 Department of Otorhinolaryngology, First People’s Hospital of Jiangxia District, Wuhan, China; 5 Department of Otorhinolaryngology, Wuhan General Hospital of the Chinese People’s Liberation Army, Wuhan, China; 6 Division of Allergy-Immunology, Department of Internal Medicine, Morsani College of Medicine, University of South Florida, Tampa, FL 33612, USA

**Keywords:** allergy, dendritic cells, T cells, RNA-seq, signal pathway

## Abstract

It has been reported that allergen dosage can impact the differentiation of dendritic cells (DCs)-mediated T cells. However, the mechanisms of such dose-dependent differentiation are poorly understood. In this study, bone marrow-derived immature DCs stimulated with Ovalbumin (OVA) of different concentrations (0, 10, 100, 1000, 10000μg/ml, respectively). DCs were then co-cultured with naïve T cells. RNA-sequencing detection and DNA methylation of DCs were performed. We show that when DCs were stimulated with low-dose (10μg/ml), 77 genes were up-regulated and 87 genes down-regulated. Most activated genes were related to ribosome synthesis and ion channel inhibition. At the medium-dose (100μg/ml), 339 genes were up-regulated and 168 genes down-regulated. Most activated genes involved cytokine synthesis and regulation of immune responses. At high-dose (10000μg/ml), 2497 genes were up-regulated and 1156 genes down-regulated. TNF signaling pathway, NF-kappa B signaling pathway, antigen processing and presentation signaling pathway were mostly up-regulated. The related co-stimulators, co-inhibitory molecules, inhibitory cytokines, negative regulating enzymes were highly expressed. The monocarbate, coenzyme, fatty acid, glucolipid, starch, sucrose and other metabolism-related signaling pathways were down-regulated. The profiles of DNA methylation and RNA synthesis of DCs varied with different doses of OVA, which serves to induce T cells to differentiate in various directions.

## INTRODUCTION

Dendritic cells (DCs) are generally deemed as crucial antigen-presenting cells in the human body and the starting point of the acquired immunity. DCs are believed to play a crucial role in inducing the differentiation of helper T cells (Th cells). Some experiments showed that the induction of the differentiation of Th cells by DCs is determined by multiple factors, such as the type and dose of antigens, the type of DCs, cytokines (IL-10, IL-12) and stimulatory factors (lipopolysaccharides, CpG) etc. [1–3]. DCs that mature under different circumstances can dictate differentiational direction of Th cells [4, 5].

It has been well reported and consensus has been reached that allergen dose can impact DC-mediated T cell differentiation. When DCs are exposed to high-dose antigen, they can induce Th0 cells into Treg and Th1 cells. Nonetheless, when DCs are subjected to low-dose antigen, they can induce Th0 to Th2 cells [6–8]. Clinically, exposure to allergens of different doses may lead to various outcomes of allergic conditions. For instance, a German multi-center study that had followed up 1,314 newborns for 20 year found that exposure to environmental dust mite allergen was proportional to the incidence rate of allergic diseases [9]. When the dust mite concentration in the environment exceeded 10μg/g, the incidence rate of dust mite allergy in families with allergy history could be as high as 5.5%. When the environmental concentration was lower than 0.1μg/g, the risk of allergic reactions was very low [10]. Tovey et al. found that the highest and lowest exposure concentrations in the quintile of dust mite in asthma were relatively low in the in children aged 0-5 years and the incidence of asthma was highest when the exposure concentrations were at 3.5-23.4μg/g [11]. The association between exposure doses and the incidence of allergic diseases might be ascribed to the aforementioned connection between antigen dose and the cell-differentiating effect of DCs in the upstream of immune responses. In the allergen-specific immunotherapy for allergic rhinitis and asthma, high concentrations of allergens were used to stimulate DCs in order to elicit the allergen-specific immune responses which will induce the conversion of T cells to Treg and Th1 cells instead of Th2 cells [12, 13]. On the other hand, low-concentration allergens tend to be ineffective with such allergen-specific immunotherapy [14].

Immature DCs (imDCs) are usually found in the immunostatic phase, expressing receptors which related to antigen recognition (such as Toll-like receptor, mannose receptor, etc.). When exposed to the antigen, imDCs will engulf the external antigens and, after experiencing a series of changes, convert into mature DCs (mDCs). These changes involve: (1) loss of receptors which mediate endocytosis, (2) over-expression of MHC-II molecules and co-stimulators, such as CD40, CD80, and CD86, etc., and (3) over-expression of CCR7 and other related receptors. These changes help DCs migrate into secondary lymph nodes, and contact with naïve T cells to present antigens [15, 16].

The studies are scanty concerning the differences in the signal molecules and cytokines produced during the DC activation and the association between such differences and the differentiating direction of the downstream T cells when imDCs are exposed to allergens of different doses. Huang et al. reported that DCs promoted the proliferation of Th1 cells, Th2 cells and Treg cells, respectively, by secreting different cytokines, such as IL-12, IL-1 and IL-2 (Th1 cells), IL-4 and IL-13 (Th2 cells), IL-10, IL-35 and TGF-β (Treg cells)[17–21]. Pfeiffer et al. demonstrated that when DCs were stimulated by low-dose of antigens, they expressed low-affinity peptide-MHC interactions and present weak signals to Th cells, thereby inducing the differentiation of Th cells into Th2 cells; when DCs were exposed to high-dose antigens, strong signals were presented and, as a result, Th cells differentiated into Th1 cells [22]. Some studies reported that exposure to allergens of different doses could alter the expression of DC surface markers and related factors, such as CD40, CD86, IL-4, IL-12, TLR, and TCR, etc., which might change differentiating direction of T cells [8, 23, 24].

DCs-induced differentiation of T cells involve multiple signaling pathways and a great many factors, including CD40, CD80, CD86, ICAM-1, CCL17, RELM-α, ERK, c-Fos, NF-kB, IRF4, p38-MAPK, KLF4, PDL2, Notch, DLL1 and DLL4, among others[25–27]. However, the mechanisms of such dose-effect relationship are poorly understood so far. In this study, we first looked into the functions of DCs after stimulation with Ovalbumin (OVA) of different doses. Then, by employing DNA methylation and RNA sequencing technology (RNA-seq), we examined the differential changes in DCs and in related signaling pathways after exposure to allergens of different doses and their impact on the differentiation of the downstream T cells.

## RESULTS

### Morphological and flow cytometric analysis of DCs

**Figure 1:**
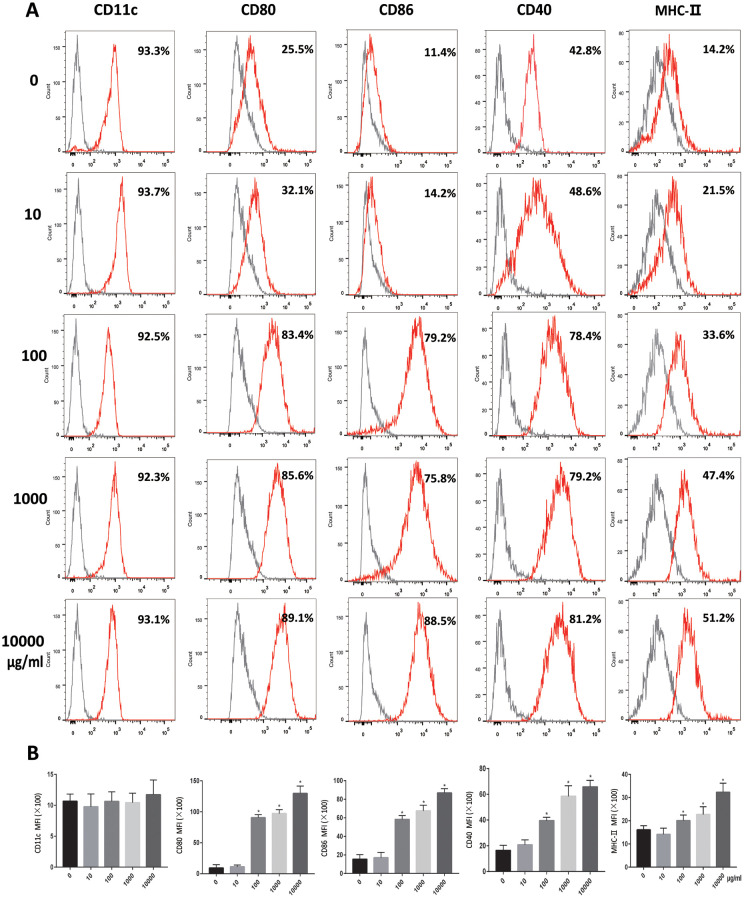
**OVA induces DCs maturation.** Immature DCs were cultured in the presence of 0, 10, 100, 1000, 10000μg/ml OVA for 24 h. (**A**). Expression of maturation-related markers on matured DCs (CD11c, CD80, CD86, CD40, MHC-II. Gray shading in histograms represents isotype controls. (**B**). Summary of the mean fluorescence intensity (MFI) of the indicated surface markers. Data indicate the mean±SD, *n* = 4. *p<0.05 represents the differences between the treatment group and the control group.

At low magnifications, a small number of cell colonies were observed on day 3. Six days later, large number of colonies were seen. At high magnifications, no dendritic branches were noticed on day 1. After day 6, the dendritic branches were evident (Supplementary Figure 1). Analyzed for the expression rate of CD11c by flow cytometry and the results showed high expression of CD11c. On day 7, flow cytometric detection showed that the expression of CD40, CD80, CD86 and MHC-II on DC increased with the OVA dose. No difference was found in the expression of CD11c among the groups. (Figure 1A, 1B).

### Flow cytometrical analysis of Th cells

**Figure 2:**
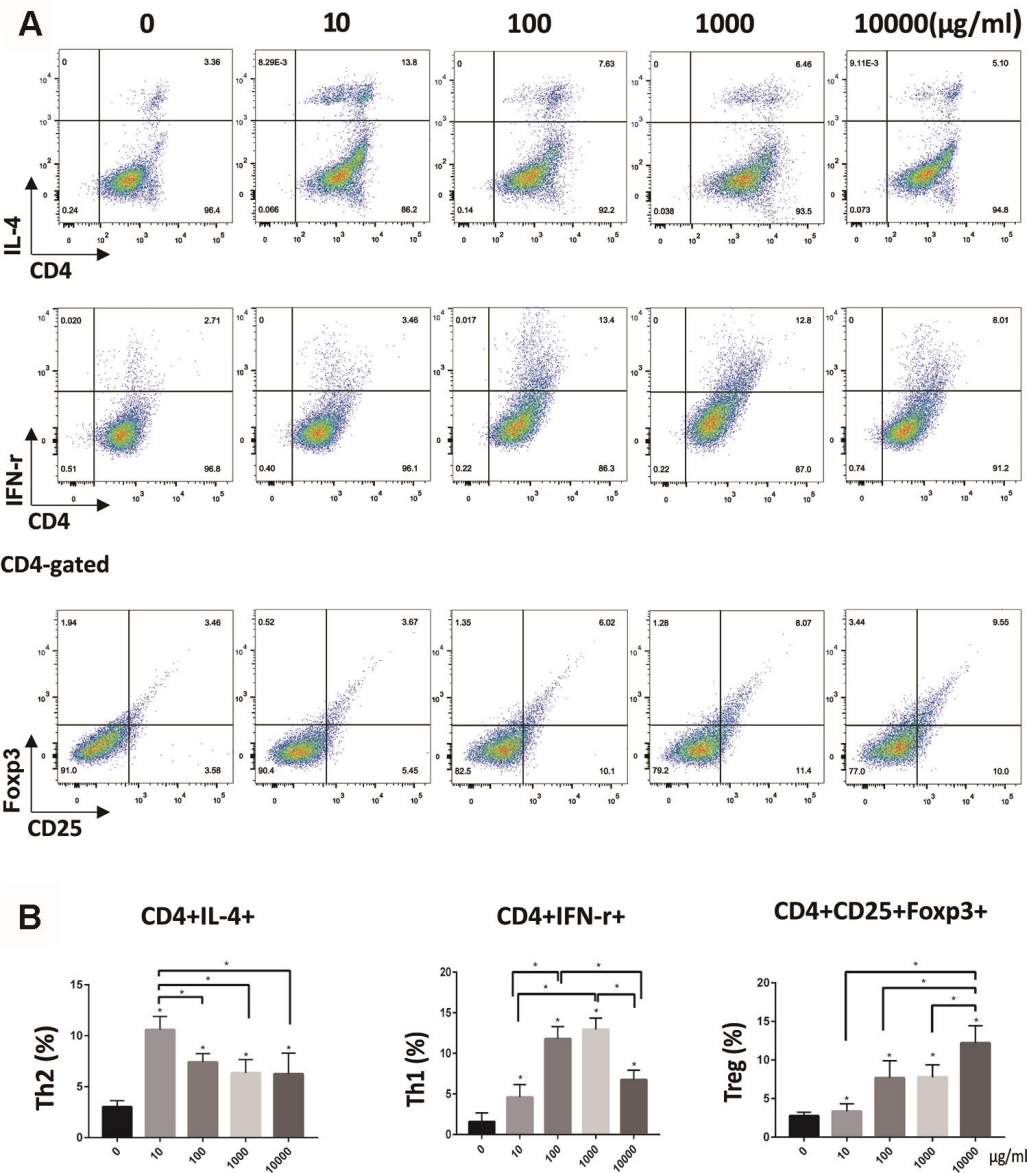
**OVA-treated DCs stimulated T-cells to differentiate into Th1, Th2 and Treg cell lines.** (**A**, **B**). Naïve CD4+ T cells were isolated using MACS from mouse systemic lymph nodes and were co-cultured for 72 h with DCs pretreated with different doses of OVA. Data indicate the mean±SD, *n* = 4. Percentage T cells is tested by flow cytometry. *p<0.05 represents the differences between the treatment group and the control group. *(P<0.05) represents the differences among the five dose groups.

Flow cytometry showed that naïve T cells could be induced to differentiate in different directions after co-cultured with DCs stimulated by OVA at different doses. In 10 DCs group, Th2 cells (CD4^+^IL-4^+^) were dominant. The 100 and 1000 DCs group, show Th1 cells (CD4^+^ IFN-γ^+^) dominance. In 10000 DCs group, Treg cells (CD4^+^CD25^+^Foxp3^+^) were dominant. There was no significant difference in the number of Th17 cells (CD4+IL-17+) among these groups (Supplementary Figure 2). Naïve T cells could not be activated when co-cultured with imDCs. (Figure 2A, 2B).

### RNA-seq and DNA methylation of DCs

Since 100 DCs group and 1000 DCs group both induce Th1 dominant, we chose the dose of 100μg/ml in the ensuing experiment. We used 0, 10, 100, 10000μg/ml OVA to stimulate DC then conduct RNA-seq and methylation analysis. The results showed that there existed a strong correlation between the gene expression level and methylation level in each group, and the experimental results were reliable and the sample selection was reasonable as shown by the Pearson correlation coefficients (> 0.8). We compared groups of different doses with the control, and conducted clustering analysis on the up-regulated and down-regulated genes by using RPKM analysis for RNA-seq and DMRs analysis for methylation, respectively. The results showed that the samples had a high homogeneity among samples in each group (Supplementary Figure 3).

**Figure 3:**
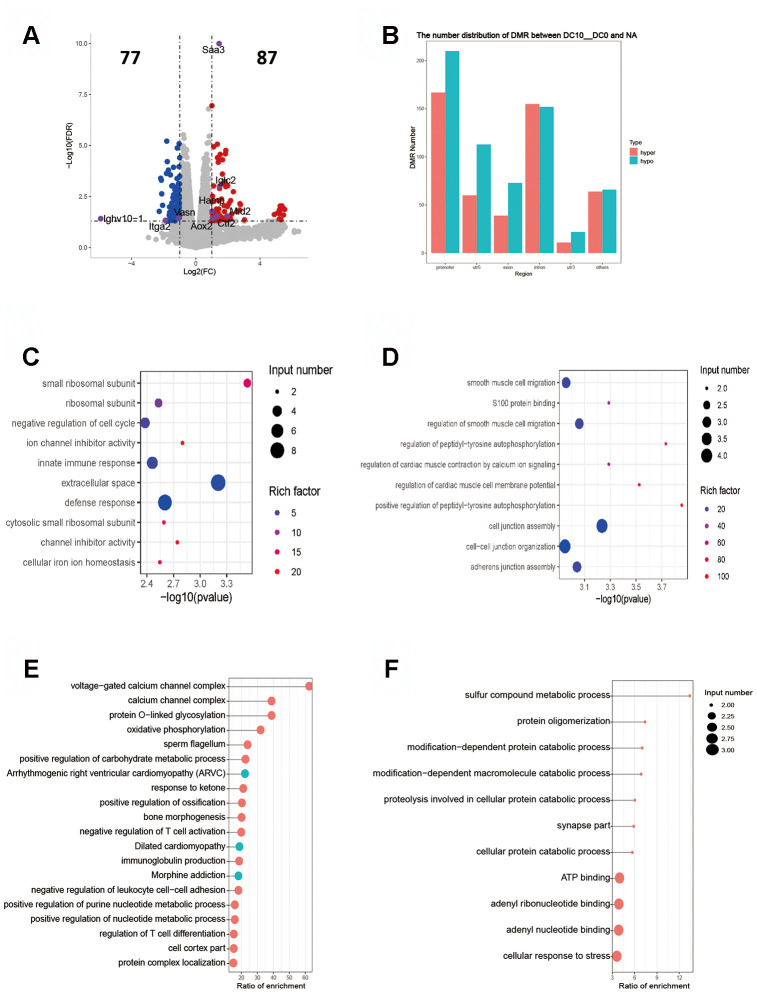
**Intergroup analysis of 10-0 DCs.** (**A**) Volcano diagram shows the genes that are differentially expressed; the gray points represent the genes that had no differential expression; the blue points represent the down-regulated genes; the red points represent the up-regulated genes. (**B**) Distribution of DMR in different regions of the genome. (**C**) Enrichment analysis of up-expressed genes. (**D**) Enrichment analysis of down-expressed genes. The horizontal axis indicates the significance of the enrichment (using -log10 (p value). The vertical axis indicates the enrichment. The dot size indicates the number of differentially expressed genes, and the dot gray scale or degree of darkness indicates the degree of rich factor enrichment. Here the 20 top most significant were selected in terms of p values. (**E**, **F**). DMR overlap gene pathway enrichment analysis. The results are shown by the scatter diagram. *n* = 3 independent experiments. (**E**). Gene body area (**F**). Promoter area (transcriptional initiation site upstream 2 kb).

**Figure 4:**
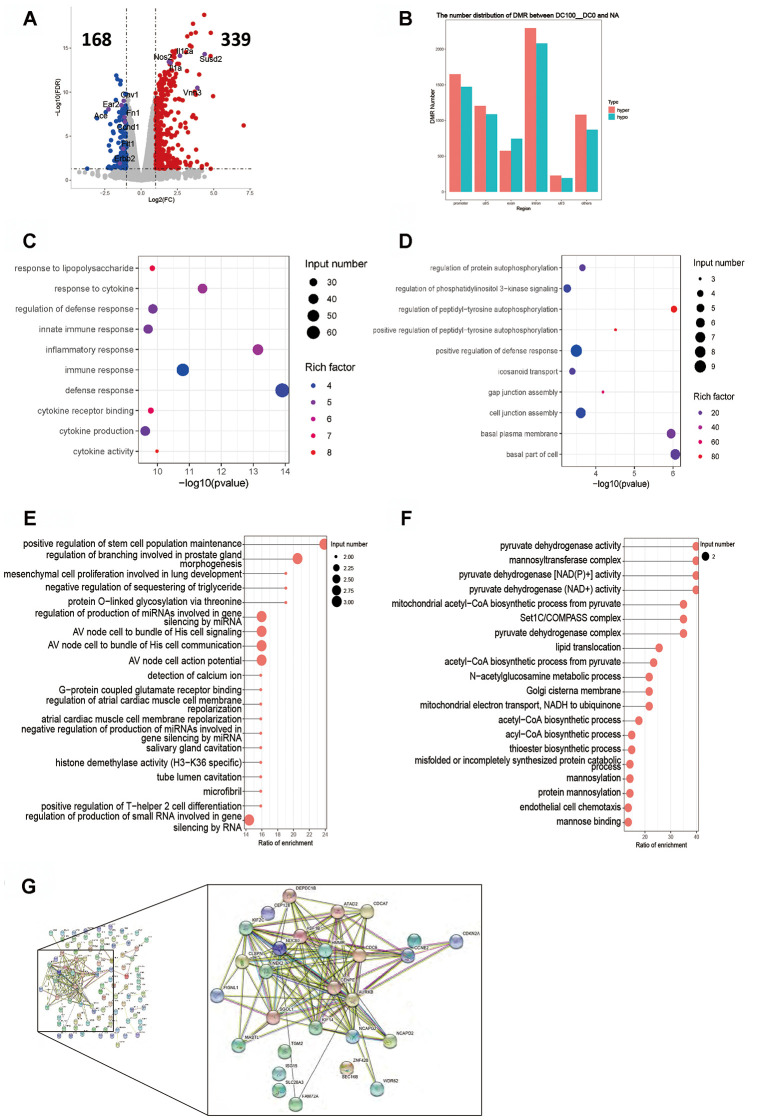
**Intergroup analysis of 100-0 DCs.** (**A**) Volcano diagram shows the genes that are expressed in the difference. (**B**) Distribution of DMR in different regions of the genome. (**C**) Enrichment analysis of up-expressed genes. (**D**) Enrichment analysis of down-expressed genes. (**E**, **F**) DMR overlapping gene pathway enrichment analysis, the results are shown by the scatter diagram. (**E**) Gene body area. (**F**) Promoter area (transcriptional initiation site upstream 2kb). (**G**) Screening for genes with significant negative correlation between RNA-seq differential gene expression and average gene methylation level. Significant negative correlation gene-protein Interaction Network Map. *n* = 3 independent experiments.

**Figure 5:**
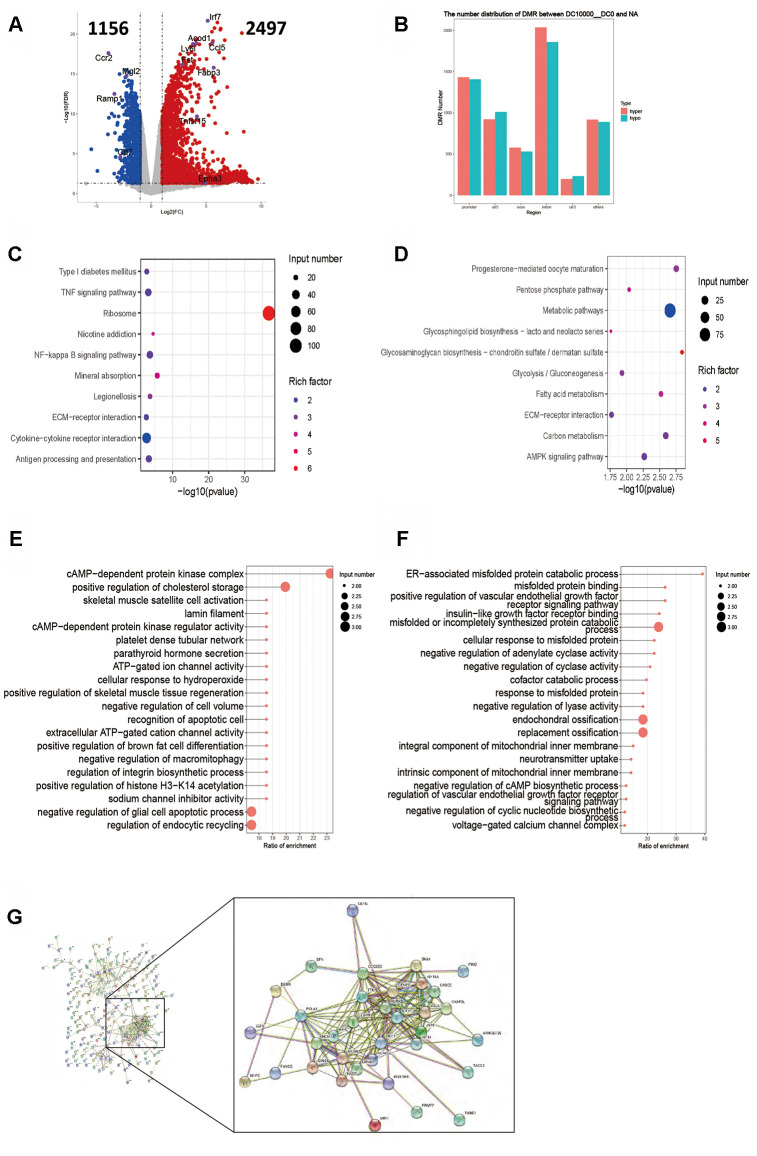
**Intergroup analysis of 10000-0 DCs.** (**A**) Volcano diagram shows the genes that are expressed in the difference. (**B**) Distribution of DMR in different regions of the genome. (**C**) Enrichment analysis of up-expressed genes. (**D**) Enrichment analysis of down-expressed genes. (**E**, **F**). DMR overlapping gene pathway enrichment analysis, the results are shown by the scatter diagram. (**E**). Gene body area. (**F**). Promoter area (transcriptional initiation site upstream 2kb). (**G**). Screening for genes with significant negative correlation between RNA-seq differential gene expression and average gene methylation level. Significant negative correlation gene-protein Interaction Network Map. *n* = 3 independent experiments.

Our study showed that DNA methylation, rather than demethylation, was the main expression profile in different regions of the genome in all dose groups (Figures 3B, 4B, 5B). In this study, the gene expression had four patterns and the methylation presented six patterns with the increasing dose of OVA. Clustering analysis showed that as the OVA dose increased, the differential gene expression level was obviously lowered or raised, and the differential methylation level showed obvious methylation or demethylation (Supplementary Figure 4). Our analysis showed that some activation signaling pathways shared by various dose groups mainly involved ribosome synthesis (e.g. Gm13423, Rps4x-ps genes), Jak-Stat signaling (e.g. Hamp, Socs1 genes), pantozoate, CoA biosynthetic and other cell-metabolism-related processes (e.g. Vnn3 gene). In the study, the differentially expressed genes were subjected to GO/KEGG enrichment analysis, and the gene body area and overlapping genes with DMRs to functional enrichment analysis. As a result, the top 10 genes with the highest significance were selected to make a plot ordered by P values. If the numbers of enriched pathways in the sample was less than 10, they were all shown in a scatter plot. The results of inter-group analyses of 10-0 DCs, 100-0 DCs, 10000-0 DCs are shown below.

### Intergroup analysis of 10-0 DCs

The RPKM results of 10 DCs group showed that 77 genes were up-regulated and 87 genes down-regulated (Figure 3A). The key up-regulated genes included Aox2, Ctf2, Hamp, Iglc2, Mid2 and Saa3. The key down-regulated genes involved Ighv10-1, Itga2, Slc8a1, Vasn. The up-regulated genes were mainly covered signaling pathways of ribosome synthesis and ion channel inhibitors, and the down-regulated genes mainly involved the signal pathway of protein auto-phosphorylation (Figure 3C, 3D). The functional enrichment analysis of methylation showed that the DMR coincidence gene (all) pathway were principally related to the positive regulation of purine nucleotide metabolism, carbohydrate metabolism and negative regulation of T-cell activation. The promoter region gene (all) pathways were mainly those involving the protein catabolic and adenyl ribonucleotide binding. The enrichment results of hypermethylated DMR coincidence genes (hyper) and hypomethylated DMR coincidence genes (hypo) are shown in Figure 3E, 3F. Genes that bore significant negative correlation between RNA expression and DNA methylation level were screened (gene body region and gene promoter region respectively). The expression of C530008M17Rik gene was negatively correlated with the average DNA methylation level of C base in the gene body region. The expression of Scgb1a1 gene was negatively correlated with the average DNA methylation level of C base in gene promoter region.

### Intergroup analysis of 100-0 DCs

The RPKM results of 100 DCs group showed 339 genes were up-regulated and 168 genes down-regulated (Figure 4A). The key up-regulated genes included Il12a, Il1a, Il23, Nos2, Susd2 and Vnn3. The key down-regulated genes involved Ace, Cav1, Ccnd1, Ear2, Erbb2, Flt1 and Fn1. The up-regulated genes were involved in the signaling pathways of cytokine synthesis, regulation of immune responses and cytokine-cytokine receptor interaction, while the down-regulated genes were mainly those which partook in the PI3K signaling pathway (Figure 4C, 4D). The functional enrichment analysis of DNA methylation exhibited that the DMR coincidence gene (all) pathway mainly covered the production of small RNA involved in RNA gene silencing and the positive regulation of stem cell population. The promoter region gene (all) pathways were mainly involved in pyruvate dehydrogenase [NAD(P)^+^] and acetyl−CoA biosynthetic process. Hypermethylated DMR coincidence genes (hyper) and hypomethylated DMR coincidence genes (hypo) are shown in Figure 4E, 4F. The genes with which their RNA expression bore significant negative correlation with DNA methylation level were screened (gene body region and gene promoter region respectively) and results showed that there existed a significant negative correlation between the expression level of 126 genes and the average methylation level of C base in the body region of genes. Protein interaction network analysis exhibited there was a strong correlation between the two events in these genes: Aurkb, Asf1b Atad2, Cep128 Clspn, Cenpe, Cdc6, Cdca7, Depdc1B, Kif2C, Nek2, Sgol1, Kif14, Hmmr and Ncapg2 (Figure 4G). No gene expression level was negatively correlated with the average DNA methylation level of C base in gene promoter region.

### Intergroup analysis of 10000-0 DCs

The RPKM results of 10000 DCs group showed that 2497 genes were up-regulated and 1156 genes down-regulated (Figure 5A). The key up-regulated genes included Acod1, Ccl5, Fabp3, Fst, Irf7, Ly6i and Tnfsf15. The key down-regulated genes included Ccr2, Cd7, Epha3, Mgl2 and Ramp1. The up-regulated genes mainly involved the TNF signaling pathway, NF-kappa B signaling pathway, antigen processing and presentation, while the down-regulated genes mainly covered on the signal pathways of carbon, fatty acids, glucolipid and sucrose metabolism (Figure 5C, 5D). The enrichment analysis showed that the DMR coincidence gene (all) pathways mainly involved cAMP-dependent protein kinase regulator activity and endocytic recycling. The promoter region gene (all) pathways principally covered the adenylate cyclase, cyclase and lyase activation, and were mainly negatively regulated. Hypermethylated DMR coincidence genes (hyper) and hypomethylated DMR coincidence genes (hypo) are shown in Figure 5E, 5F. The genes for which RNA expression was significantly negatively correlated with DNA methylation level (gene body region and gene promoter region respectively) were screened and the results revealed that there existed a significant negative correlation between the expression level of 253 genes and the average DNA methylation level of C base in the body region. There were good relationship of the genes including Aurkb, Cdc25c, Cenpe, Clspn, Casc5, Ckap2L, Kif2C, Kif14, Kif18A, Ttk, H1f0, Mcm7, Gins1, Nasp, Lmnb2, Ncapg2, Prc1, Oip5, Sgol1 and Ska1 in protein interaction network analysis (Figure 5G). No gene expression level bore negative correlation with the average DNA methylation level of C base in gene promoter region.

### Functional analysis of DCs

**Figure 6:**
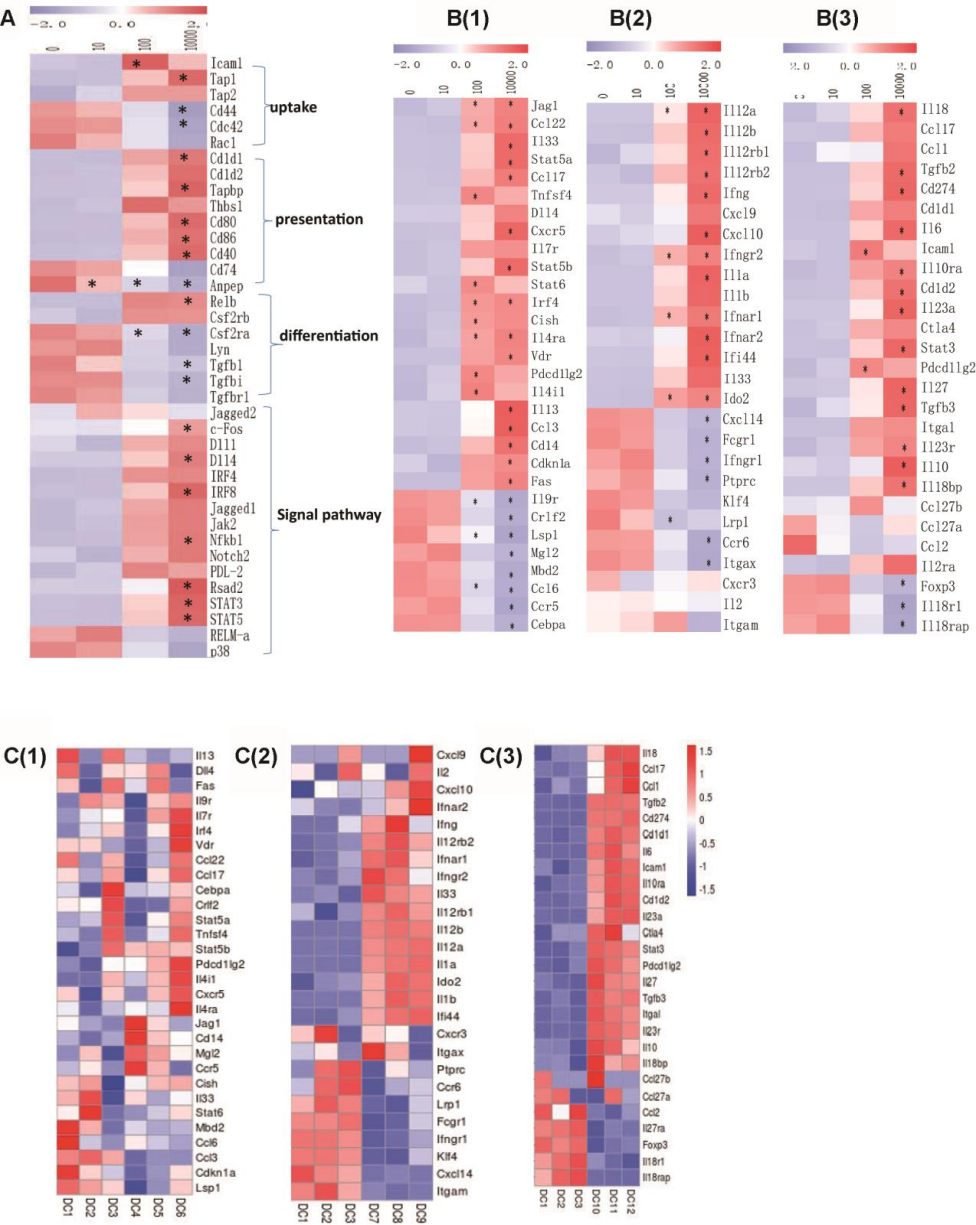
(**A**) The RPKM value of DC function genes in each dose group in each concentration group. **B(1)** The RPKM value of the gene induced Th2 cells differentiation in each concentration group. **B(2)** The RPKM value of the gene induced Th1 cells differentiation in each concentration group. **B(3)** The RPKM value of the gene induced Treg cells differentiation in each concentration group. **C(1)** The RPKM value of the gene induced Th2 cells differentiation in 10-0 group. **C(2)** The RPKM value of the gene induced Th1 cells differentiation in 100-0 group. **C(3)** The RPKM value of the gene induced Treg cells differentiation in 10000-0 group. DC1/2/3 represents 0ugml DC4/5/6 represents 10ug/ml. DC7/8/9 represents 100ug/ml DC10/11/12 represents 10000ug/ml. Data indicate the mean±SD *p<0.05 represents the differences between the treatment group and the control group. *n* = 3 independent experiments.

We analyzed the activation of the functional genes in different groups according to RPKM. It was found that with the OVA dose increasing, the up-taking genes Icam1, Tap1 and Tap2 were up-regulated, while Cd44, Cdc42 and Rac1 were down-regulated. The antigen-presenting genes Cd1d1, Cd1d2, Tapbp, Thbs1, Cd80, Cd86 and Cd40 were up-regulated, while Cd74 and Anpep were down-regulated. The differentiation-related genes Relb and Csf2rb were up-regulated, while Csf2ra, Lyn, Tgfb and Tgfbr1s were down-regulated. The signal-transducing genes Jagged1, Jagged2, c-Fos, Dll1, Dll4, Irf4, Irf8, Jak2, Nfkb, Notch, Pdl2, Rsad2, Stat3 and Stat5 were up-regulated, while RELM-α and p38 were down-regulated (Figure 6A). As to the functional genes of different subgroups of Th cells, no obvious differences were found in Th2 cells-related genes including Il4, Il13, Dll4, Fas, Il9r, Il7r, Irf4, Vdr, Ccl22, Ccl17, Cebpa, Crlf2, Stat5, Stat6, Tnfsf4, Pdcd1lg2, Cxcr5, Jag1, Cd14, Mgl2, Ccr5, Cish, Il33, Mbd2, Ccl6, Ccl3, Cdkn1a and Lsp1 between in 10-0 DCs groups. Th1 cells-related genes including Cxcl9, Cxcl10, Ifna, Ifng, Il12, Il2, Ifnar1, Ccl4, Il1, Ido2 and Ifi44 were up-regulated, and Cxcr3, Itgax, Ptprc, Ccr6, Fcgr1, Ifngr1, Klf4, Cxcl14, Itgam were down-regulated between in 100-0 DCs groups. Most Treg cells-related genes including Il18, Ccl17, Ccl1, Tgfb, Cd274, Il6, Icam1, Il23, Ctla4, Stat3, Pdcd1lg2, Il27, Itgal, Il10 were up-regulated between in 10000-0 DCs groups (Figure 6B, 6C).

## DISCUSSION

In this study, we cultured imDCs from murine bone marrow mononuclear cells, stimulated them with OVA of different doses, and then co-cultured the mature DCs with naïve T cells. Although the DCs from different sources were functionally various, the response patterns are similar when exposed to antigens of gradually-increasing dose. We selected DCs derived from bone marrow-derived mononuclear cells, because they are relatively stable in terms of number and function. Meanwhile, availability of multiple studies on Mono-DCs allowed for easy comparison among studies [28]. The results showed that the differentiation of naïve T cells, when cultured with DCs stimulated by different doses of OVA, differs substantially in terms of differentiation direction. When DCs were stimulated with low dose of OVA (10μg/ml), and co-cultured with naïve T cells, CD4^+^IL-4^+^expression was relatively up-regulated (suggesting differentiation towards Th2 cells). When DCs were stimulated with medium dose of OVA (100μg/ml), and co-cultured with naïve T cells, CD4^+^ expression was relatively elevated (indicating differentiation towards Th1 cells). When DCs were stimulated with high dose of OVA (10000μg/ml), and co-cultured with naïve T cells, CD4^+^CD25^+^Foxp3^+^expression was relatively higher (indicative of differentiation towards Treg cells). Pfeiffer et al. also reported that low-dose OVA could induce Th2 response, while higher-dose OVA could elicit Th1 response [22]. By stimulating DCs with TSST-1 of different doses, Rothoeft et al. also found that, when stimulated with 100 ng/ ml TSST-1, Th cells mainly produced IFN-γ, when stimulated with 0.1 ng/ ml TSST-1, Th cells mainly secreted IL-4 [29]. To exclude the influence of lipopolysaccharides, we tested the lipopolysaccharides concentration in OVA and found that its amount were marginal (Supplemental Table 1). Meanwhile, in order to observe the effect of the duration of DC-T cells co-culture on the differentiation of T cells, we extended the co-culture time to 72 hours and detected the markers on T cells. The result showed that there were no significant differences in the proportions of T cells subgroups between 3 day and 1 day co-culture (Supplementary Figure 5). These findings led us to speculate that DCs stimulated by OVA at different doses can induce naïve T cells differentiating towards different directions, and the differentiation is mainly dose but not time-dependent.

We then determined whole-gene DNA methylation and conducted RNA-seq in DCs stimulated with OVA of different doses. It was found that not many genes were activated between in 10-0 DCs groups (The RPKM results showed that only 77 genes were up-regulated and 87 genes down-regulated). The activated signaling pathways and most of the activated genes were related to ribosome synthesis and ion channel inhibition, but not to the immune response and Th2 activation. DMR results also showed that signaling pathways involving regulation of purine nucleotide metabolism, carbohydrate metabolism were up-regulated, while the pathways concerning T cell activation were down-regulated in 10 DCs group. Kamath et al. proposed a "default" model of Th2 differentiation and the "default" pathway, i.e., DCs can activate Th2 response preferentially through specific pathogen recognition receptors [30]. Pletinckx et al. also theorized that DCs had a natural mild Th2 response tropism. They found that very few genes were activated when DCs were stimulated with the Th2 specific agonist TNF or VSG, and the activated genes were non-specific genes of inflammation. Twenty four activated genes identified in their study were consistent with the genes found in our research [31] (Supplementary Figure 6A). Noble et al. demonstrated that calcium channel activation is beneficial to the polarization of Th1, while calcium channel inhibition tends to activate the Th2 pathway. In this study, low-dose OVA stimulation mainly activated the pathways of calcium ion channel inhibition, which was consistent with Noble’s results [32]. Therefore, we are led to assume that DCs have a natural tendency to induce Th2 response and DCs can induce Th2 differentiation even if a small amount of allergen is present in the environment.

In 100 DCs group, DNA methylation and RNA synthesis were significantly enhanced compared with the 0 DCs group and 10 DCs group. RPKM results showed that 339 genes were up-regulated and 168 genes down-regulated. GO/KEGG enrichment analysis showed that genes involving cytokine synthesis and regulation of immune response and signaling pathway of cytokine-cytokine receptor interaction were mostly up-regulated, while PI3K signaling pathway was down-regulated. Pletinckx et al. reported that when DCs induced Th1 polarization of T cells, the activated genes were significantly more than the genes of Th2 polarization (a total of virtually 5,000 genes were activated in Th1 polarization of DCs, and only 160 genes were activated during Th2 polarization). As aforementioned, the signaling pathways of cytokine synthesis were activated and we found that most of the genes related to Th1 induction were up-regulated, and, in particular, IDO2, IL-12, CD14 and IFN-γ were highly expressed. Vander et al. found that, during DCs-induced Th1 polarization, HMGN1 R848, MAPKs, IRF3, IRF7 and IFN-α were significantly up-regulated, which is also consistent with our results [33] (Supplementary Figure 6B). Nonetheless, we also found that some genes involving Th1 polarization were down-regulated (such as CXCR3, ITGAX, CCR6, etc.). We speculated that at certain concentrations, although the differentiation of DCs may overall goes to a certain direction (polarization), there is still a certain mechanism of positive regulation and negative feedback, which precisely controls the direction immune reaction. There should be a balance between positive and negative feedbacks, which dictates the expression of the Th1-related genes. Briefly, when exposed to a certain dose of allergen, the immune response of DCs is significantly enhanced. DCs could induce Th1 polarization, with most Th1-related genes being activated and, at the same time, some negative feedback remaining.

The changes in DNA methylation level and RNA level are further enhanced in 10000 DCs group. The number of activated genes was about 100 times more than 10 DCs group, and about 10 times than 100 DCs group (RPKM results showed that 2497 genes were up-regulated and 1156 genes were down-regulated). The GO/KEGG enrichment analysis showed that TNF signaling pathway, NF-kappa B signaling pathway, antigen processing and presentation signaling pathway were mainly up-regulated. The related co-stimulators, co-inhibitory molecules, inhibitory cytokines, negative regulating enzymes were highly expressed. The carbon, fatty acids, glucolipid, sucrose metabolism and other metabolism-related signaling pathways were down-regulated. Vendelova et al. reported that the major up-regulated genes of tolerance DCs derived from mouse mononuclear cells included SOCS2, CD83, CD150, CD200, CD274, ALDH1A2, etc., which was in line with our results [34]. It was reported that imDCs could induce tolerance, that is, imDCs processed and presented antigens without co-stimulatory molecules, leading to the T cell dysfunction or deficiency. However, in the past few years, fully mature tolerance-regulating dendritic DCs were found in various environments and they highly expressed co-inhibitory molecules (PD-L1, PD-L2) and secreted inhibitory factors (IL-10, PGE2, TNF-α, TGF-β, IL-27), and induce T cells towards Treg. In current study, we found that DCs were highly activated, with most Treg related genes being up-regulated (IL-18, CCL17, CCL1, TGF-β, CD274, IL-6, ICAM-1, IL-23, CTLA4, STAT3, PDCD1LG2, IL-27, ITGAL, IL-10, et al). That is, DCs not only showed high activated status under high concentration OVA stimulation, but also related co-stimulatory molecules, co-suppressor molecules, inhibitory cytokines and negative regulatory enzymes were highly expressed. Therefore, it can be inferred that DC tolerance is a highly active behavior. By altering the potential activation state of the initial DC, DCs develop tolerance itself and induce the differentiation of downstream Th0 cells into Treg cells.

## CONCLUSIONS

We found some common activated signaling pathways of DCs when stimulated by OVA of different doses, including ribosome synthesis (e.g. Gm13423, Rps4x-ps gene), JAK-STAT signaling pathway (e.g. Hamp, Socs1 gene), pantozoate, CoA biosynthetic and other cell-metabolism-related processes (e.g. Vnn3 gene), etc.. More importantly, DCs can have many different DNA methylation and RNA synthesis when stimulated by OVA of different doses, which perform the function of inducing T cells to different polarization. The differential gene expression of DCs in DNA methylation level, RNA synthesis level and signaling pathways involved might provide potential targets for intervening DCs, thereby changing the differentiation direction of the downstream T cells. Meanwhile, it laid a foundation for further exploration of the core points of DC for dose recognition.

## MATERIALS AND METHODS

### Animals

Six-week-old female BALB/c mice (SPF) were purchased from Beijing HFK Bioscience Co., Ltd., Beijing, China. All experiments were performed in strict accordance with the recommendations of the Guidelines for the Care and Use of Laboratory Animals of the National Institutes of Health, China. The animal study was reviewed and approved by the Committee on the Ethics of Animal Experiments of Huazhong University of Science and Technology, Wuhan, China

### Experimental materials

The following antigens, cytokines and antibodies were used in the study: OVA (≥98%, Sigma-Aldrich, St. Louis, USA); Granulocyte-macrophage Colony Stimulating Factor (GM-CSF), Recombinant Mouse IL-4 (r-Mu IL-4) (R&D, Minneapolis, USA). Isotype control antibodies (Abs) (IgG1, IgG2a or IgG2b), anti-mouse CD11c PerCP-Cy5.5, anti-Mouse CD80 PE-Cy7, anti-Mouse CD86 PE, anti-Mouse CD40 APC, anti-mouse MHC-II FITC, anti-Mouse CD4 FITC, anti-mouse CD62L PE, anti-mouse CD25 BV421, anti-mouse IL-4 PerCP-Cy5.5, Anti-Mouse IFN-γ PerCP-Cy5.5, Anti-Mouse Foxp3 APC, Anti-Mouse CD3e (eBioscience, San Diego, USA); Mouse Naïve CD4^+^ T cell isolated kit (Stemcell, Carlsbad, USA). Red Blood Cell Lysis Buffer (Beyotime, Shanghai, China)

### DC propagation

Bone marrow DCs were generated as previously described1. Briefly, bone marrow was removed from the femurs and tibias of BALB/c mice. Following red cell lysis, cells were grown in RPMI 1640 medium supplemented with 20% FBS, GM-CSF(20ng/ml), IL-4(5ng/ml) at a density of 1×10^6^ cell/ml. Cells were incubated at 37° C in 5% CO_2_ and fed with fresh media every two days. Half of the medium was removed and replaced with fresh differentiation medium every other day. Repeated pipetting was used to collect loosely adherent cells for later analysis.

### Preliminary identification of DCs

The morphological changes of the cells in the culture system were observed dynamically under an optical inverted-phase contrast microscope on daily basis. The purity of DCs was flow cytometrically detected. On the 6^th^ day after the culture, the culture plate was taken out from the incubator, the medium was gently blown, and the non-adherent cells were blown up and collected. After centrifugation washing, the flow antibody CD11c were added according to the instructions and detected by the machine.

### Effects of different doses of OVA on the maturity of DCs

After cultured for 6 days, the imDCs were stimulated with OVA of different concentrations (0, 10, 100, 1000, 10000μg/ml, respectively) for 24h. DCs were divided into 5 groups: 0 DCs group (stimulated with 0μg/ml OVA), 10 DCs group (10μg/ml OVA stimulated), 100 DCs group (100μg/ml OVA stimulated), 1000 DCs group (1000μg/ml OVA stimulated) and 10000 DCs group (10000μg/ml OVA stimulated). After the suspending cells were harvested, the CD11c, CD40, CD80, CD86, and MHC-II were added and the samples were detected on a flow cytometer.

### Co-culture of DCs and CD4^+^T cells

The Naïve CD4^+^ T cells were isolated by using magnetic-activated cell sorting (MACS) from mouse cervical, axillary, inguinal and mesenteric lymph nodes, and flow cytometric antibodies CD4 and CD62L were added to detect the initial T cell purity. The imDCs were stimulated with 0, 10, 100, 1000, 10000μg/ml OVA. After incubation for 24h, the mDCs were seeded (1×10^5^ cells/well in 1ml) into a complete medium with naïve T cells (1×10^6^cells/well) for 3 days in 48-well plates. After the co-culture, CD4^+^ T cells were stained for expression of CD4, IL-4, IFN-γ, CD25, Foxp3. All these cell populations were analyzed by using Cytoflex S Flow Cytometer (Beckman Coulter, USA) and the data were analyzed by using a CytExpert software package.

### RNA-seq of DCs

On the sixth day, OVA solutions of different doses were added to the DCs taken from mouse bone marrow, and the concentration of OVA in each hole was 0, 10, 100, and 10,000μg /ml, respectively. Three samples were tested for each group, and the culture lasted for 24 hours. After the collection of suspension cells, the cells, in each group, were separated and resuscitated in 0.5ml Trizol buffer (Takara, Dalian, China). RNA-seq was performed (Wuhan Kangce Co., LTD., China). Trizol extraction is a reliable method for obtaining high-quality total RNA. RNA-seq library was constructed by Illumina TruSeq RNA Sample Prep Kit version 2 (Illumina, San Diego, CA) and RNA was sequenced by Illumina HiSEquation 2000 platform (Illumina). Intergroup analysis: 10-0 DCs (10 DCs group compare with 0 DCs group), 100-0 DCs (100 DCs group compare with 0 DCs group), 10000-0 DCs (1000 DCs group compare with 0 DCs group).

### DNA Methylation analysis of DCs

After the DCs were collected and centrifuged as aforementioned, the cells of each group were separated, and methylation analysis and sequencing were carried out (Wuhan Kangce Technologies Co., LTD., China). Genomic DNA was extracted by Cetyl trimethyl ammonium bromide method and EZ DNA Methylation-Gold™ Kit ^+^ accel-ngs ® Methyl-seq DNA Library Kit were employed to construct the Bisulfite sequencingLibrary before high-throughput sequencing. The sequencing was performed on Illumina HiSeq 2000. By using Base Calling software, the original imaging data collected by Illumina HiSeq sequencing was converted into sequence data, i.e. FASTQ format, to obtain the most original sequence data file. Clean reads were mapped to reference genome with Bismark (v0.20.1). Differentially methylated regions (DMRs) are regions in which methylation levels are significantly different among samples and may play important roles in the regulation of gene expressions. DMR distribution in different regions of the genome was statistically analyzed. Mfuzz of R package was employed to determine the concentration gradient sequence. Mfuzz is based on the fuzzy clustering theory and analyzes the gradient sequences of the same cell lines according to the expression patterns of differentially expressed genes. It also clustered samples on the basis of average methylation levels of the samples. Heat map was generated by using TIGR Multiexperiment Viewer software. Overlapping gene set enrichment analysis was conducted against Gene Ontology/ Kyoto Encyclopedia of Genes and Genomes (GO/KEGG) database. On the basis of the correlation between gene expression and average methylation, the genes with Cor_P value<0.05 and Cor value<0, were identified and the results indicated that the gene expression level was negatively correlated with the methylation level, and thus expression of the genes might be regulated by their methylation. Enrichment analysis and protein interaction analysis were performed on the selected genes.

### Statistical analysis

Data were processed using GraphPad Prism 7.0 statistical software, and the data were expressed as mean ± standard deviation. The differences between groups in findings of the flow cytometry were compared by one-way analysis of variance (ANOVA). P-value<0.05 was considered statistically significant. The differences in Reads per Kilobase per Million Reads (RPKM) expression and methylation results in RNA-seq results were analyzed by ANOVA. The enrichment analysis in the GO/KEGG results were hypergeometrically tested against KEGG Pathway and GO Term, with P-Value<0.05 as the standard.

### Ethics approval

All experiments were performed in strict accordance with the recommendations of the Guidelines for the Care and Use of Laboratory Animals of the National Institutes of Health, China. The protocol was approved by the Committee on the Ethics of Animal Experiments of Huazhong University of Science and Technology, Wuhan, China.

## Supplementary Material

Supplementary Figures

Supplementary Table 1
