# Supplementary material for: Different doses of ovalbumin exposure on dendritic cells determine their genetic/epigenetic regulation and T cell differentiation
Source: Aging (Albany NY). 2020 Nov 24;12(24):25432–51. doi: 10.18632/aging.104145 (PMC7803576; doi:10.18632/aging.104145)
Supplement: Supplementary Figures [file aging-12-104145-s001.pdf]

SUPPLEMENTARY FIGURES

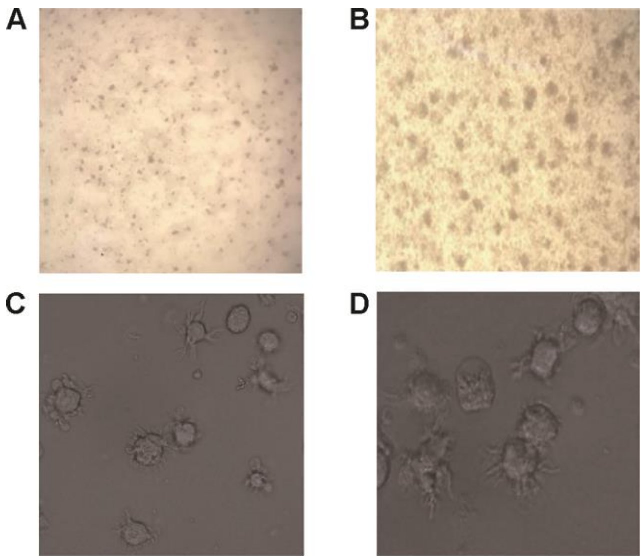

Supplementary Figure1. Identification of DC cells. The morphological of the DCs (×100). (**A**) 3rd day, a small number of cell colony form (**B**) 6th day, a mass of cell colony. The morphological of the DCs (×400). (**C**) 4th day, DCs were irregular in morphology and exhibited multiple dendritic processes (**D**) 6th day, DC cells form typical dendritic branches

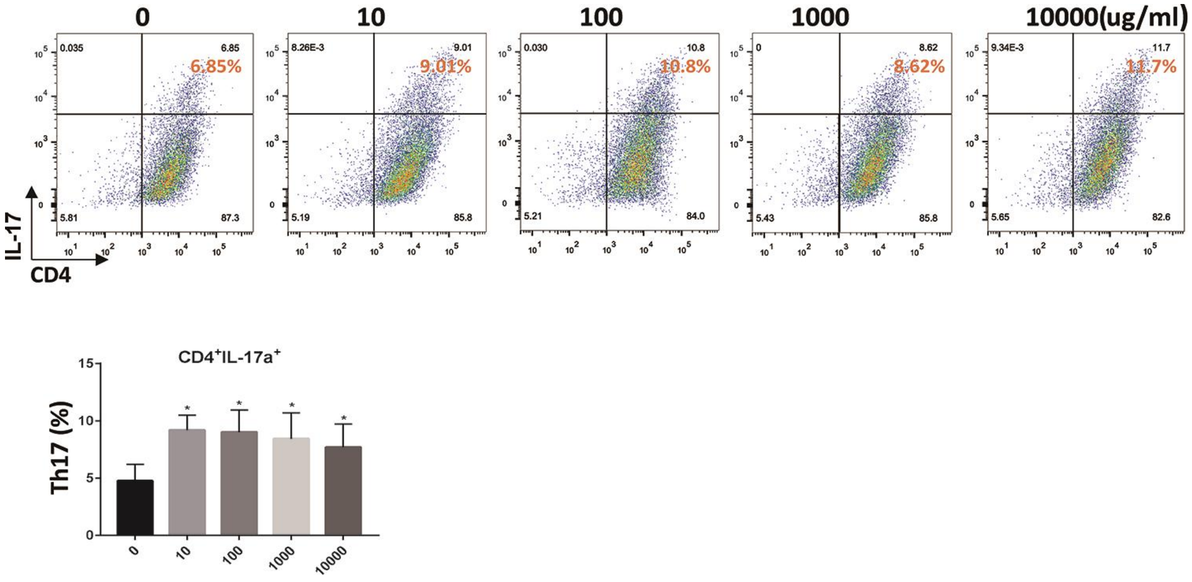

Supplementary Figure 2. OVA-treated DCs stimulated T-cells to differentiate into Th17. Naïve CD4+ T cells were isolated using MACS from mouse systemic lymph nodes and were co-cultured for 72 h with DCs pretreated with different doses of OVA. Error bars represent standard deviations. ∗$p<0.05$ represents the differences between the treatment group and the control group.

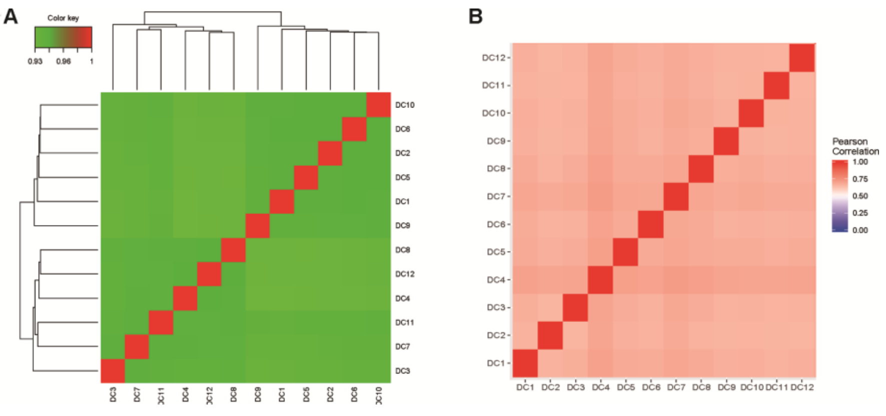

Supplementary Figure 3. Sample biology repeated correlation test. (**A**) Hierarchical clustering of sample gene expression levels; color scales from green to red represent lower inter-sample correlations to higher inter-sample correlations obtained by gene expression levels, respectively. (**B**) Using the methylation site detection results of the samples to perform correlation between samples. The closer the correlation coefficient is to 1, the higher the similarity in the methylation pattern between samples. n = 3 independent experiments.

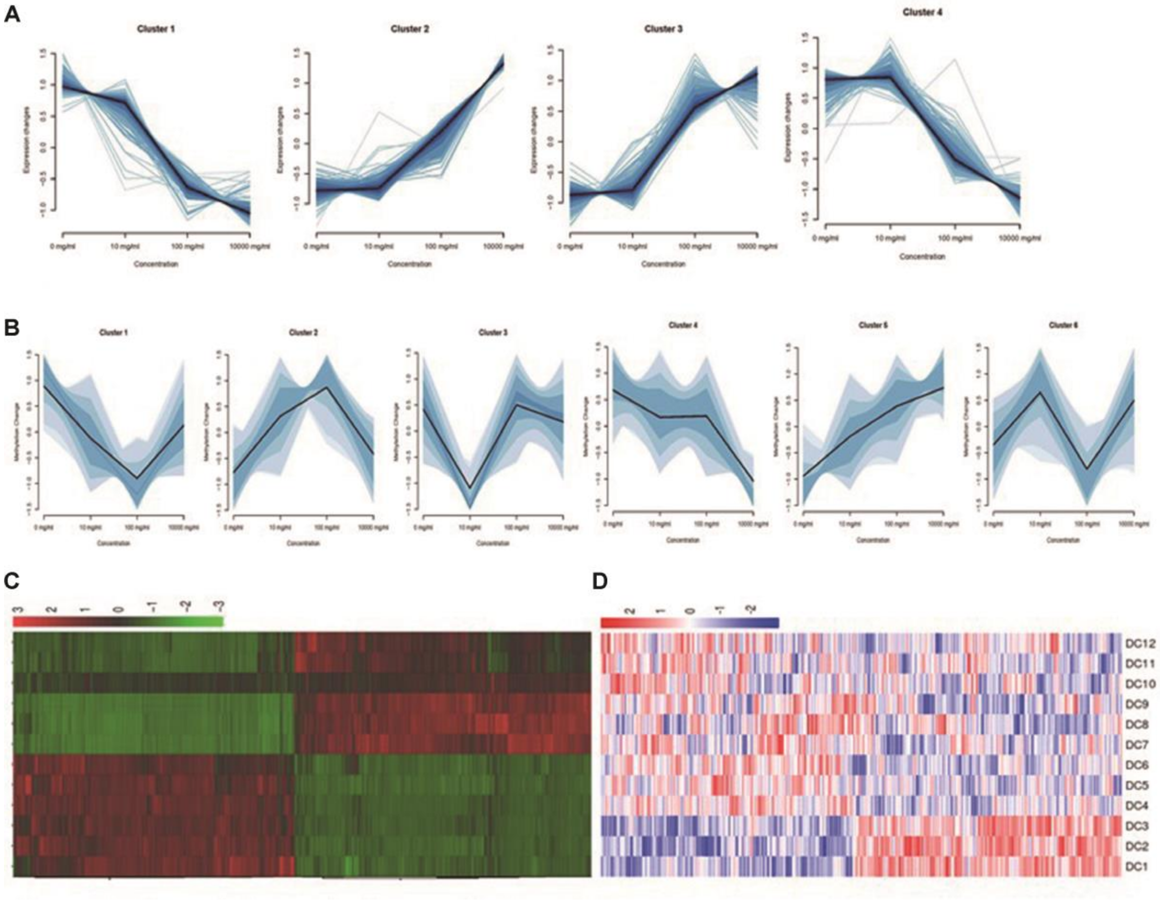

Supplementary Figure 4. Concentration gradient analysis. (**A**) There were four patterns of gene expression in DCs as OVA concentration gradually increased; (**B**) There were six patterns of methylation in DCs as OVA concentration gradually increased Cluster analysis of differentially expressed genes and differentially methylated genes: (**C**) The expression of differentially expressed genes was significantly down-regulated or up-regulated as the concentration of OVA gradually increased; (**D**) The expression levels of differentially methylated genes were significantly methylated or demethylated as the concentration of OVA gradually increased. n = 3 independent experiments. DC1/2/3 represents 0 ug/ml DC4/5/6 represents 10 ug/ml DC7/8/9 represents 100 ug/ml DC10/11/12 represents 10000 ug/ml .

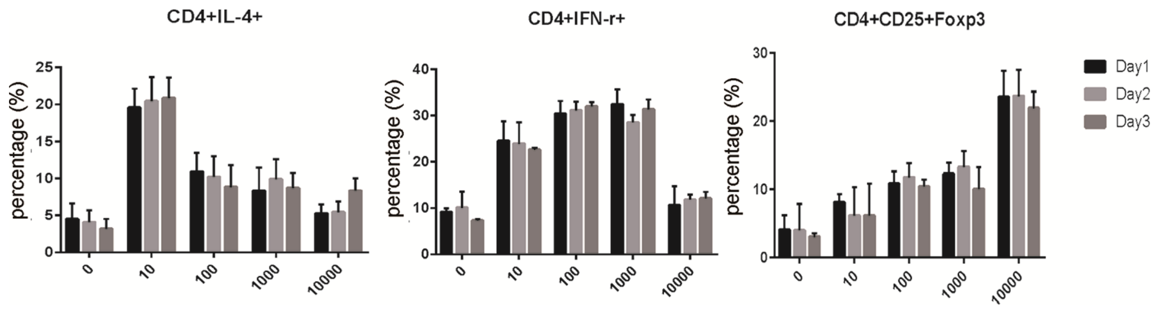

Supplementary Figure 5. To stimulate DCs for longer periods of time(24, 48, 72h), and co-cultured with T cells. n = 6 independent experiments.

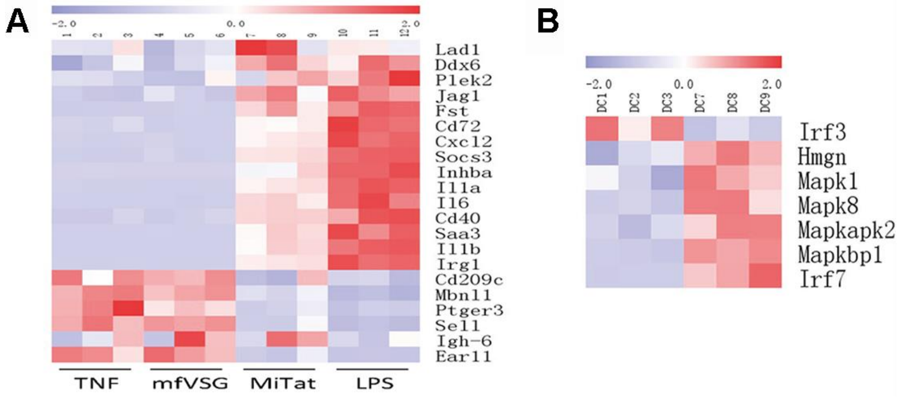

Supplementary Figure 6. (**A**) The gene were activated by TNF, mfVSG, MiTat, lipopolysaccharides are consistent with our results. (**B**) In the 100 DCs group, HMGN, MAPKs and IRF7 were also up-regulated.
