# Supplementary material for: Different doses of ovalbumin exposure on dendritic cells determine their genetic/epigenetic regulation and T cell differentiation
Source: Aging (Albany NY). 2020 Nov 24;12(24):25432–51. doi: 10.18632/aging.104145 (PMC7803576; doi:10.18632/aging.104145)
Supplement: Supplementary Table 1 [file aging-12-104145-s002.pdf]

SUPPLEMENTARY TABLE

Supplementary Table 1. LPS concentrations in different doses of OVA.

OVA	LPS
10mg/ml	2.917pg/ml
100mg/ml	4.104pg/ml
1000mg/ml	19.548pg/ml
10000mg/ml	63.143pg/ml
